# Supplementary material for: Implementing Innovative Approaches to Improve Health Care Delivery Systems for Integrating Communicable and Non-Communicable Diseases Using Tuberculosis and Diabetes as a Model in Tanzania
Source: Int J Environ Res Public Health. 2023 Aug 29;20(17):6670. doi: 10.3390/ijerph20176670 (PMC10487244; doi:10.3390/ijerph20176670)
Supplement: Supplementary file 1 [file ijerph-20-06670-s001.zip › Supplementary SI.pdf]

Table S1: Assessment tool: Nurse Officer (Cluster 1).

	Parameter for assessment	Grading	Score
**1**	Empower others HCW in the facility	a. Evidence of Ongoing Empowerment of all HCWs **(4-points)**<br>b. Evidence of Train all staff but utilize only few CME sessions **(3-points)**<br>c. Evidence of Empower only those working in TBDM Clinic **(2-points)**<br>d. Evidence of Empower only facility in charge/ administrators **(1-point)**<br>e. No evidence of any empowerment to other staff **(0 point)**	
**2**	Trained staff at the assigned distant health facility	a. Evidence of Training staff and ongoing practice in the assigned facility (**2-points)**<br>b. Evidence of training but no ongoing practice in the facility **(1-point)**<br>**c.** No evidence of training in the facility **(0 point)**<br>d. Not Applicable	
**3**	Trainer demonstrates knowledge/ ability/ skills/ on pharmacovigilance	a. Knowledge of pharmacovigilance and Evidence of practice **(2-points**)<br>b. Knowledge of pharmacovigilance and no Evidence of practice **(1-point**)<br>c. No insight on pharmacovigilance **(0-point)**	
**4**	Trainer demonstrates knowledge/ ability/ skills/ on IPC	a. Knowledge of IPC and Evidence of practice **(2-points)**<br>b. Knowledge of IPC and no Evidence of practice **(1-point)**<br>c. No insight on IPC **(0-point)**	
**5**	Prepared nursing care plan to patients with TB and associated comorbidity/ multi morbidity	Minimum required number is 15 i.e. 100% (15points)<br>a. $^{X}/_{15}$<br>Not applicable	
**6**	Quality of Generated care plans	a. Excellent **(4-points)**<br>b. Very Good **(3-points)**<br>c. Good **(2-points)**<br>**d.** Satisfactory **(1-points)**<br>e. Poor below **(0-point)**<br>f. Not applicable	
**7**	Average Facility Performance reflecting cluster 1-Contribution	a. X/10<br>**b.** Not applicable	

Table S2: Assessment tool: Medical doctors/specialists (Cluster 1/mentors).

	Parameter	Grading	Score
1	Empower others HCW in the facility	1. Evidence of Ongoing Empowerment of all HCWs (**4 points**)<br>2. Evidence of Train all staff but utilize only few CME sessions (**3points)**<br>3. Evidence of Empower only those working in TBDM Clinic (**2 points**)<br>4. Evidence of Empower only facility in charge/ administrators (**1point**)<br>5. No evidence of any empowerment to other staff (**0 point**)	
2	Trained staff at the assigned distant health facility	1. Evidence of Training staff and ongoing practice in the assigned facility (**2 points**)<br>2. Evidence of training but no ongoing practice in the facility (**1 point**)<br>3. No evidence of training in the facility (**0 point**)<br>4. NA (for Regional Mentors)	
3	Trainer demonstrates knowledge/ ability/ skills/ on pharmacovigilance	1. Knowledge of pharmacovigilance and Evidence of practice **(2points**)<br>2. Knowledge of pharmacovigilance and no Evidence of practice (**1point**)<br>3. No insight on pharmacovigilance (**0 point**)	
4	Screening for DM in TB populations	$^{X}/_{10}$	
5	Screening for TB in DM population	$^{X}/_{10}$	
6	Screened for other multimorbidity in dual TBDM population	1. **Very good**=Evidence of screening Hypertension, Malnutrition and HIV status **(2 points**)<br>**2. Good=** Evidence of screening at least one multimorbidity **(1 point)**<br>3. **Poor** = No evidence for screening multimorbidity **(0 point)**	
7	Interpretation of finding for clinically relevant action	a. **Very good**=Evidence of screening height and weight and interpretation of BMI (**2 Points**)<br>b. **Good**= Evidence of screening Height and Weight with no interpretation (**1 point)**<br>c. **Poor** = No evidence for screening BMI **(0 point)**	
8	HbA1c utilization practice	a. $^{X}/_{10}$<br>b. Not Applicable	
9	Management of patients with dual TBDM comorbidity	a. Very good=Evidence of re-arrangement clinic for attending patients with dual TBDM and other multimorbidity **(2 points)**<br>b. Good= Partial evidence of re-arrangement for attending patients with dual TBDM and other multimorbidity (**1 point)**<br>c. Poor = No evidence available **(0 point)**<br>d. Not Applicable	

10	Trainer demonstrated knowledge/ ability/ skills/ on IPC	a. Knowledge of IPC and Evidence of practice **(2points)**<br>b. Knowledge of IPC and no Evidence of practice **(1point)**<br>c. No insight on IPC (**0 point)**	
11	Average Facility Performance reflecting mentors- Contribution	**$^{X}/_{10}$**	

Table S3: Assessment tool_3. Performance of the cluster 1/cluster 2 health care facilities.

	Parameters	Grading	Score
1	Facility in charge is aware and shows ownership in improving TBDM services (Any innovation/ Best practice for improving TBDM services)	a) Highly Oriented and committed (strong sense of ownership **(3points)**<br>**b)** Oriented and shows some commitment **(2 points)**<br>c) Was not oriented but demonstrates willingness to improve the services /Oriented but not willing **(1 point)**<br>d) Not oriented and not willing **(0 point)**	
2	A trained facility organizes clinics for managing patients with TB	a) Presence of TB clinic **(2points)**<br>b) Available plan to establish the clinic in the near future **(1points)**<br>c) No TB clinic, no plan to establish the clinic in the near future **(0 point)**	
3	A trained facility organizes clinics for managing patients with DM	a) Presence of DM clinic **(2points)**<br>b) Available plan to establish the clinic in the near future **(1point)**<br>c) No DM clinic, no plan to establish the clinic in the near future **(0point)**<br>d) Not Applicable (depends on the level/capacity of the facility)	
4	A trained facility organizes clinics for managing those clients with dual TB and DM and multi morbidities.	a) Presence of TBDM clinic **(2points)**<br>b) Available plan to establish the clinic in the near future **(1point)**<br>c) No TBDM clinic, no plan to establish the clinic in the near future **(0 point)**<br>d) Not applicable depends on the level of facility	
5	A trained facility organizes referral of clients with dual TB and DM and multi morbidities.	a) Presence of evidence of referred patients **(2 points)**<br>**b)** Demonstrates an organized referral mechanism (tracking referred client) **(1 point)**<br>c) No evidence of referred client or organized referral mechanism **(0 point)**	
6	Pharmacovigilance	a) Presence of evidence of pharmacovigilance practice to patient	

		who have experienced severe adverse events **(2 points)**<br>b) Demonstrate knowledge of pharmacovigilance but no evidence of practice **(1 point)**<br>c) Absence of evidence and knowledge and practice of pharmacovigilance **(0 point)**	
**7**	A trained facility considered IPC particularly the clinics arrangements (Documentation/posters of IPC for the best practice available at the HF)	**a)** Presence of evidence of IPC practice **(2 points)**<br>b) Demonstrate knowledge of IPC but no evidence of practice **(1 point**)<br>c) Absence of evidence and knowledge and practice of IPC **(0 point)**	
**8**	A trained Facility screen for DM in TB population and actively use DM screening register in TB unit	a) Demonstrate evidence of screening all TB patients for DM **(2 points)**<br>b) Demonstrate Partial evidence of screening TB for DM **(1-point)**<br>c) No evidence of screening TB for DM **(0-point)**	
**9**	A trained facility screen for TB in DM population and actively use TB presumptive register.	a) Demonstrate evidence of screening all DM patients for TB **(2 points)**<br>b) Demonstrate Partial evidence of screening DM for TB **(1-point)**<br>**c)** No evidence of screening TB for DM **(0-point)**<br>d) Not Applicable	
**10**	Other comorbidities such as hypertension, BMI, are screened and documented	a) Demonstrate evidence of screening TB or DM or dual TBDM patients for other multi morbidities **(2- points)**<br>b) Demonstrate Partial evidence of screening TB or DM or dual TBDM patients for other multi morbidities **(1-point)**<br>c) No evidence of screening TB or DM or dual TBDM patients for other multi morbidities **(0 point)**	
